# Carbon Nanomaterials: Efficacy and Safety for Nanomedicine

**DOI:** 10.3390/ma5020350

**Published:** 2012-02-21

**Authors:** Takuya Yamashita, Kohei Yamashita, Hiromi Nabeshi, Tomoaki Yoshikawa, Yasuo Yoshioka, Shin-ichi Tsunoda, Yasuo Tsutsumi

**Affiliations:** 1 Laboratory of Toxicology and Safety Science, Graduate School of Pharmaceutical Sciences, Osaka University, 1-6 Yamadaoka, Suita, Osaka 565-0871, Japan; E-Mails: t-yamashita@nibio.go.jp (T.Y.); yamasita@phs.osaka-u.ac.jp (K.Y.); nabeshi@phs.osaka-u.ac.jp (H.N.); tomoaki@phs.osaka-u.ac.jp (T.Y.); 2 Laboratory of Biopharmaceutical Research, National Institute of Biomedical Innovation, 7-6-8 Saito-Asagi, Ibaraki, Osaka 567-0085, Japan; E-Mail: tsunoda@nibio.go.jp; 3 The Center for Advanced Medical Engineering and Informatics, Osaka University, 1-6 Yamadaoka, Suita, Osaka 565-0871, Japan; 4 Laboratory of Biomedical Innovation, Graduate School of Pharmaceutical Sciences, Osaka University, 1-6 Yamadaoka, Suita, Osaka 565-0871, Japan

**Keywords:** nanomedicine, carbon nanomaterials, fullerenes, carbon nanohorns, carbon nanotubes, drug delivery, nano safety science

## Abstract

Carbon nanomaterials, including fullerenes, carbon nanohorns, and carbon nanotubes, are increasingly being used in various fields owing to these materials’ unique, size-dependent functions and physicochemical properties. Recently, because of their high variability and stability, carbon nanomaterials have been explored as a novel tool for the delivery of therapeutic molecules including peptide and nucleic acid cancer drugs. However, insufficient information is available regarding the safety of carbon nanomaterials for human health, even though such information is vital for the development of safe and effective nanomedicine technologies. In this review, we discuss currently available information regarding the safety of carbon nanomaterials in nanomedicine applications, including information obtained from our own studies; and we discuss types of carbon nanomaterials that demonstrate particular promise for safe nanomedicine technologies.

## 1. Introduction

Advances in nanotechnology have led to the recent development of many nanomaterials, including nanoscale silica particles, titanium dioxide nanoparticles, and carbon nanomaterials [1,2,3,4]. Nanomaterials, which are generally classified as materials with feature sizes smaller than 100 nm, have remarkably impacted various fields of study because of the desirable properties (e.g., enhanced electrical conductivity, tensile strength, and chemical reactivity) imparted by their increased surface area per unit weight compared with that of their bulk-scale counterparts [5,6]. Nanomaterials are already being applied in electronics [1], foods [2], and cosmetics [3]. Furthermore, in basic research for development of new drugs, nanomaterials are expected to open novel avenues for the treatment of human diseases owing to their unique physicochemical properties [4]. 

Carbon nanomaterials, including fullerenes, carbon nanohorns (CNHs), and carbon nanotubes (CNTs), have been used as carriers in drug delivery and other applications [7]. Carbon nanomaterials with carbon cage and graphene structures have many technological advantages such as facile modification by functional groups [8,9,10], high carrier capacity [11,12], high chemical stability [13,14], and feasibility of incorporating both hydrophilic and hydrophobic substances [15,16] (Table 1). These characteristics, which are essential for the development of drug-delivery carriers, make carbon nanomaterials promising for nanomedicine applications. 

**Table 1:** Basic physicality of carbon nanomaterials.

	Fullerenes	CNHs	CNTs
Year of discovery	1985	1998	1991
Discoverer	H.W. Kroto R.F. Curl R.E. Smalley	S. Iijima	S. Iijima
**Size**			
Diameter Length	1 nm -	2–4 nm 40–70 nm	0.4–70 nm 1 µm–2.5 mm
Shape	sphere	horn	fiber
Practical use	Cosmetics Lubricity agent Semiconductor	Fuel battery	Semiconductor Car parts Sports goods

Though highly promising, these carbon nanomaterials are in an early phase of development. Therefore, information regarding their safety is not sufficient for the development of medically sound and nontoxic technologies. Because nanomaterials’ physicochemical properties often differ substantially from those of their bulk counterparts, as mentioned above, there are concerns that carbon nanomaterials may exhibit unexpected side effects. In addition, recent reports have shown that pristine CNTs might induce mesothelioma-like lesions in mice, similar to those induced by asbestos [17,18,19]. On the other hand, Muller *et al.* showed that pristine CNTs induce no mesothelioma formation in a 2-year *in vivo* study [20]. Therefore, more information about the safety of nanomaterials needs to be collected. 

In this review, we discuss currently available information about the safety of carbon nanomaterials for nanomedicine applications, including information obtained from our own previous studies. We also discuss types of carbon nanomaterials that demonstrate particular promise for safe nanomedicine technologies.

## 2. Utility of Carbon Nanomaterials for Nanomedicine

### 2.1. Fullerenes

Fullerenes have attracted considerable attention in various fields of science [21]. Fullerenes are composed entirely of carbon in the form of a hollow sphere, ellipsoid, or tube. Spherical fullerenes are also referred to as buckyballs. An important property of the fullerene molecule is their high symmetry. There are 120 symmetry operations, such as rotation around an axis and reflection in a plane. Fullerenes belong to the class of inorganic nanoparticles and show high bioavailability due to their small size (~1 nm). Owing to their small size, fullerenes can penetrate various tissues and organelles that materials with submicron size cannot penetrate. For example, Foley *et al.* reported that fullerenes can cross the COS-7 cell membrane and bind to the mitochondria [22], demonstrating that fullerenes have utility as intracellular carriers. Furthermore, fullerenes’ capability to act as drug-delivery carriers for low-molecular-weight compounds and oligonucleotides has been demonstrated [23]. For example, conjugates composed of fullerenes and paclitaxel have exhibited the potential to provide slow release of the drug and have exhibited significant anti-cancer activity in cell cultures [24]. Moreover, Maeda-Mamiya *et al.* reported effective gene delivery *in vivo* using water-soluble fullerenes [25]. In that study, conjugates consisting of cationic tetraamino fullerenes and an insulin-gene-expressing plasmid complex were injected intravenously into C57/BL6 mice. Insulin gene expression was detected in the lung, liver, and spleen. Plasma insulin levels in the insulin gene group of mice were significantly higher than those in a control group. Both of these studies demonstrate that fullerenes may act as drug- and gene-delivery carriers. Furthermore, because fullerenes are strong anti-oxidants, they have been used as neuroprotective [26,27] and anti-inflammatory agents [28]. Thus, if fullerenes can be controllably manipulated, they could be used to treat various diseases. 

### 2.2. CNHs/CNTs

CNHs and CNTs based on the structure of graphene are also regarded as drug-delivery carriers [29,30]. CNHs and CNTs are differentiated from each other by their shape and size (Table 1). Furthermore, CNHs and CNTs can be classified as possessing either single- or multi-walled (SW or MW) structures. This review cites several reports on the use of SWCNHs and SWCNTs as drug-delivery carriers. SWCNHs have plenty of inner spaces. Through these holes, various molecules such as low-molecular-weight compounds or nucleic acids can enter the hollow interior of the SWCNHs. SWCNHs also can regulate the sustained release of drugs from their interior for drug-delivery applications. For example, the release rate of cisplatin (CDDP), a chemotherapy drug that can be incorporated into oxidized SWCNHs, has been regulated by controlling solvent composition. The release of CDDP from the SWCNHs is slower in water and a culture medium than in phosphate-buffered saline, and the CDDP released from SWCNHs in the former solvent effectively kills human lung-cancer cells [11]. 

SWCNTs also have been demonstrated to be amenable for drug delivery. SWCNT-siRNA conjugates have been efficiently transported to human T-cells and primary cells, which are inert to commercially available liposome-based nonviral vectors, and have silenced a specific gene in those cells [31]. In cancer therapy, SWCNT-based tumor-targeted drug-delivery system (DDS) has already been developed by several investigators [32,33]. SWCNT-anticancer-drug conjugates also have shown higher efficacy in suppressing tumor growth than clinical anticancer drugs alone in various cancer models [32,33]. These therapeutic effects were induced by accumulation of the conjugates in tumor. Collectively, these results clearly indicate the potential applications of SWCNHs and SWCNTs in cancer-targeted drug delivery and sustained release [34]. 

### 2.3. Suitable Modification of Carbon Nanomaterials for DDS 

Accumulation at a targeted location is important in DDS. Carbon-nanomedicine-based cancer treatment systems generally function by means of either active targeting or passive targeting. In active-targeting DDS for cancer treatment, the search for cancer-specific targets is important. SWCNTs modified with antibodies, folate, arginine-glycine-aspartic acid (rgd) peptide, and epidermal growth factors have been useful for active targeting of tumor tissue [35,36,37,38]. Ruggiero *et al.* reported that antibody-modified SWCNTs accumulate in tumor tissue in a murine xenograft model of human colon adenocarcinoma [39]. However, these anti-cancer effects are not enough to enable drug development because these targets do not express specifically in tumor. In recent years, novel targets have been identified by using “-omics” approaches such as proteomics, genomics, and metabolomics [40,41,42]. Proteomics-based analysis is currently a promising approach for identifying biomarker proteins for use in drug development because these proteins directly regulate the onset and progression of diseases. However, proteomics-based analysis can yield many potential candidate biomarker proteins that are over- or under-expressed in diseased tissues, and these candidates must be efficiently screened to identify appropriate targets. Toward this end, we have developed an “antibody proteomics system” that facilitates the screening of biomarker proteins from many candidates by rapid preparation of cross-reacting antibodies using phage antibody library technology. The system is an efficient method for screening tumor-related biomarker proteins to identify novel targets [43]. 

In passive-targeting DDS for cancer treatment, improvement of drug retention in blood is important because the reticulo-endothelial system and kidney work as the barrier against foreign particles *in vivo*. Covalent conjugation of polyethylene glycol (PEG) to a carrier’s surface, referred to as “PEGylation” is a promising strategy to improve retention of various nanomaterials in the blood [44]. PEGylation can prolong the plasma half-life and alter the tissue distribution of the nanomaterial conjugates compared with their non-PEGylated forms, which typically clear the body through the reticulo-endothelial system *in vivo*. The extended circulating lifetime of PEGylated conjugates in blood induces an enhanced permeability and retention effect, which is based on the leaky nature of tumor blood vessels, resulting in increased delivery of the conjugates to tumor tissue. As an example, Yang *et al.* investigated the long-term *in vivo* biodistribution of nanoscale graphene sheets functionalized with PEG and systematically examined the potential toxicity of graphene over time [45]. On the other hand, from the aspect of effectivity and safety, CNTs kinetics is important for drug development. Singh *et al.* describes the pharmacokinetic parameters of intravenous administered functionalized SWCNTs relevant for various therapeutic and diagnostic applications [46]. It shows that functionalized (water-soluble) SWCNTs can, in fact, be excreted via the renal route. In summary, to obtain highly effective and nontoxic carbon nanomaterial DDS for cancer treatment, it is necessary to control three factors: (1) size; (2) the ability to target the molecules to tumors and (3) clearance through the reticulo-endothelial system and kidney. 

### 2.4. Other Application of CNTs in Medicine

As mentioned above, CNTs have been explored as a novel tool for the delivery of therapeutic molecules including peptide, nucleic acid and cancer drugs. On the other hand, certain types of CNTs have been reported to possibly help cancer diagnosis and other application [47,48]. Photoacoustic imaging proposes higher spatial resolution and permits deeper tissues to be imaged compared with most optical imaging techniques. Zerda *et al.* [47] showed plain SWCNTs conjugated with cyclic Arg-Gly-Asp (RGD) peptides can be used as a agent for photoacoustic imaging of tumors. This report indicates SWCNTs is possibly useful for cancer diagnosis. Additionally, Tosun *et al.* suggested collagen conjugated SWCNTs show the potential for enhanced electrical activity. These SWCNTs have been shown positive *in vitro* biocompatibility results offering further evidence that SWCNT-based materials have an important role in neuronal regeneration [48]. Neurodegenerative disorders including Parkinson’s and Alzheimer’s diseases, amyotrophic lateral sclerosis are rapidly increasing as the population ages. The field of nanomedicine promises revolutionary advances to the diagnosis and treatment of devastating human diseases [48]. 

## 3. Safety of Carbon Nanomaterials

### 3.1. Hazard Assessment

Carbon nanomaterials are among the most promising nanomedicines. However, information about the safety of carbon nanomaterials is still fragmentary, and ensuring their safety is of utmost importance to protect human health. In this section, we focus on the safety of CNTs specifically, because some studies have reported that CNTs have higher toxicity than fullerenes and CNHs [49]. 

Parameters such as structure, size distribution, surface area, surface chemistry, surface charge, and agglomeration state as well as purity of the samples, have considerable impact on the reactivity of CNTs. Some studies have reported that certain types of SW or MW CNTs are cytotoxic and genotoxic *in vitro*, so public concern about the potential risk of CNTs to human health has risen [50,51,52]. In fact, recent reports have indicated that certain types of CNTs might induce mesothelioma-like lesions in mice, in a manner similar to that observed for mesothelioma induced by asbestos [53,54,55]. Takagi *et al.* showed that intraperitoneally administered pristine MWCNTs induce mesothelioma in the p53 (+/−) mouse carcinogenesis model, probably due to the MWCNTs’ resemblance to asbestos in size and shape and to their biopersistency [17]. Poland *et al.* also observed asbestos-like pathogenic behavior of long pristine MWCNTs associated with their needle-like fiber shape and established a structure-activity relationship based on the length of the MWCNTs [19]. These studies revealed that the propensity of long MWCNT fibers to produce inflammation and fibrosis in the peritoneal cavity is similar to, or greater than, that of long asbestos fibers. In contrast, neither short asbestos fibers nor short tangled MWCNTs cause any significant inflammation [19]. These results suggest that physical properties, such as length, diameter and physico-chemical properties, might impact the safety of pristine CNTs [56]. However, these studies were based on the administration of extremely high doses of MWCNTs via peritoneal injection. In contrast, Shvedova *et al.* showed that pristine MWCNTs enhanced acute inflammation and pulmonary injury with delayed bacterial clearance after aspiration or inhalation of MWCNTs [57,58]. In the future, it is needed to examine the study relevant to the human occupational exposure situation. 

There are a few reports that examine the mechanisms of CNT toxicity [59,60,61]. One important underlying factor that influences the safety of long fibers is the failure of macrophage cells to completely enclose them. This failure, termed incomplete or “frustrated” phagocytosis, can induce inflammation [19]. Migliore *et al.* showed that long rigid MWCNTs appear to form fiber-like aggregates or structures that are too long to be phagocytosed by macrophage cells, thus resulting in reactive oxygen species (ROS) production [62,63], which contributes to NACHT domain-, leucine-rich repeat-, and pyrin domain-containing protein 3 (NLRP3) activation [64,65]. Palomaki *et al.* demonstrated that the NLRP3 inflammasome was essential for long, needle-like CNTs and asbestos to induce IL-1β secretion [65]. Moreover, it was noted that CNT-induced NLRP3 inflammasome activation depended on ROS production [65]. Clarification of the mechanism of inflammation induced by CNTs might lead to the development of safe carbon nanomedicine technologies.

Although some studies have reported concern about the safety of CNTs as mentioned above, other studies have reported that certain types of CNTs are safe materials for nanomedicine. Yang *et al.* demonstrated that after intravascular injection of pristine SWCNTs, mice did not show stress or symptoms of abnormality, such as lethargy, anorexia, or changes in body weight [66]. Furthermore, Wick *et al.* showed that the cytotoxicity of purified rope-like agglomerated SWCNTs was lower than that of well-dispersed SWCNTs [67]. In addition to the fiber-like structure of CNTs, the amount of metal contaminants such as iron or nickel found in the CNTs may contribute to the nanomaterials’ potential carcinogenicity by accelerating the generation of ROS [68,69,70]. Moreover, in preparation for drug development, it is important to examine the influence of the oxidative debris on CNTs [14,71,72,73]. It is an emergent and key point during purification and functionalization of carbon nanostructure. These studies suggest that the safety of CNTs is determined not only by physical properties but also by a wide variety of factors such as method of administration, dispersability, and presence of metal contaminants. How much these factors contribute to the safety of CNTs remains unknown, however. We believe that the information obtained by these safety studies might be useful for ensuring the safety of CNTs.

### 3.2. Biological Behavior of CNTs 

Evaluation of *in vivo* kinetics is important for assessing the safety of nanomedicine technologies. In this section, studies about the behavior of CNTs in the body are described. Ruggiero *et al.* showed that intravascularly injected pristine SWCNTs favor liver accumulation and hepatobiliary excretion over kidney accumulation and renal excretion [39]. In addition, several studies have investigated pulmonary effects subsequent to instillation, aspiration, and inhalation of pristine SWCNTs [57]. These reports showed that short and small tangles of SWCNTs that deposit subpleurally migrate to the pleural space and exit in the flow of pleural fluid through the stomata, where they follow the lymphatic drainage to the mediastinal lymph nodes [60,74]. In addition, long carbon nanotubes also reach the pleural space but cannot negotiate the stomata, and so they are retained in the pleural space, where they cause inflammation and potentially long-term disease [60,74]. 

Kagan *et al.* showed that hypochlorite and reactive radical intermediates of the human neutrophil enzyme myeloperoxidase catalyze the biodegradation of carboxylated SWCNTs *in vitro*, in neutrophils and to a lesser degree in macrophages [75]. Importantly, the biodegraded nanotubes do not generate an inflammatory response when aspirated into the lungs of mice [75]. In addition, Liu *et al.* have reported that the biodurability of SWCNTs depends on surface functionalization [76]. Based on these findings, strategies for mitigating the pro-inflammatory effects of these nanomaterials in occupational settings may be developed. 

Furthermore, information about toxicokinetics (absorption, distribution, metabolism and elimination) also should be obtained for the development of safe CNTs. 

### 3.3. Development of Safe Nanomaterials 

We have discussed above how nanomaterials can serve as useful nanomedicine technologies and have also highlighted the importance of considering these nanomaterials’ safety for such applications. In this section, we examine the current status of the development of safe and effective nanomaterials for nanomedicine. In our own studies, we have established relationships between the physical properties and safety of CNTs. Our data showed that pristine thin MWCNTs and SWCNTs do not induce genetic damage *in vitro* and inflammation *in vivo* [77]. These data indicate that physical properties such as particle length and width might influence the safety of CNTs. In addition, Nagai *et al.* suggested the large-diameter or tangled MWCNTs are less toxic, less inflammogenic, and less carcinogenic than untangled MWCNTs [56]. These results suggest that control of the diameter of CNTs could be used to develop CNTs that are safe for human health. 

Furthermore, in addition to being critically important for the detection of biomolecules, the surface properties of nanomaterials also can modulate the materials’ safety. Recent studies have shown that functionalization of CNTs with carboxyl or amino surface groups can affect the CNTs’ toxicity [78]. Thus, regulation both of particle size and of surface properties is considered important for research leading to the development of safe nanomedicine technologies.

In fact, our previous study showed that nanoscale silica particles, which we expected to be useful as drug-delivery carriers, display different intracellular localization compared with submicron- and micro-scale silica particles and induce a greater cytotoxic response to mouse macrophage cell line [79]. We have also shown that nanoscale silica particles induce certain cellular responses, such as ROS generation and DNA damage to human keratinocyte cell line [80]. These results indicate that particle size could influence the silica particles’ safety for applications in nanomedicine. In addition, we have shown that surface modification of silica particles with functional groups, such as amino or carboxyl groups, suppresses toxic biological effects of silica particles including inflammatory responses and ROS production [81]. A recent study demonstrated that nanomaterials become coated with serum proteins and induce different cellular responses from intact particles by binding to proteins [82]. In addition, different surface characteristics, such as surface charge, influence the binding affinities of proteins to nanomaterials [82,83]. In fact, Gasser *et al.* showed that functionalization of MWCNTs have the potential to alter the MWCNTs blood plasma protein coating in biological systems [84,85]. These results indicate that particle size or surface properties of carbon nanomaterials can affect their safety, and that control of these physical properties can be used to advance the development of safe nanomaterials.

## 4. Conclusions

The unique physicochemical properties of carbon nanomaterials allow them to incorporate targeting ligands, chemotherapeutic drugs, and many other therapeutic agents that have great potential for cancer-targeted therapy. However, owing to the large number of factors that influence the kinetics of drug release from nanomaterials, as well as their safety for human health, insufficient information is available on these two important subjects. Factors that influence the safety of and kinetics of drug release from nanomaterials include their shape, length, and dispersability, as well as the presence of metal contaminants. A detailed understanding of the pharmacological and toxicological properties of carbon nanomaterials, as well as a balanced evaluation of their risks and benefits to human health, is required before they can be recommended for routine clinical use. We believe that a detailed safety analysis of carbon nanomaterials will be invaluable for the design of safe nanomedicine technologies.
